# *APOE* Molecular Spectrum in a French Cohort with Primary Dyslipidemia

**DOI:** 10.3390/ijms23105792

**Published:** 2022-05-21

**Authors:** Yara Abou Khalil, Oriane Marmontel, Jean Ferrières, François Paillard, Cécile Yelnik, Valérie Carreau, Sybil Charrière, Eric Bruckert, Antonio Gallo, Philippe Giral, Anne Philippi, Olivier Bluteau, Catherine Boileau, Marianne Abifadel, Mathilde Di-Filippo, Alain Carrié, Jean-Pierre Rabès, Mathilde Varret

**Affiliations:** 1 Laboratory for Vascular Translational Science (LVTS), Paris Cité University and Sorbonne Paris Nord University, INSERM, F-75018 Paris, France; yara.abou-khalil@inserm.fr (Y.A.K.); catherine.boileau@inserm.fr (C.B.); marianne.abifadel@usj.edu.lb (M.A.); jean-pierre.rabes@aphp.fr (J.-P.R.); 2 Laboratory of Biochemistry and Molecular Therapeutics (LBTM), Faculty of Pharmacy, Pôle Technologies-Santé (PTS), Saint-Joseph University, Beirut 1004 2020, Lebanon; 3 CarMen Laboratory, INSERM U1060, INRAE U1397, Université Lyon 1, F-69921 Oullins, France; oriane.marmontel@chu-lyon.fr (O.M.); mathilde.di-filippo@chu-lyon.fr (M.D.-F.); 4 Department of Biochemistry and Molecular Biology, Hospices Civils de Lyon, F-69500 Bron, France; sybil.charriere@chu-lyon.fr; 5 Department of Cardiology, Toulouse Rangueil University Hospital, UMR 1295 INSERM, F-31400 Toulouse, France; jean.ferrieres@univ-tlse3.fr; 6 Cardiovascular Prevention Centre, Centre Hospitalo-Universitaire, F-35033 Rennes, France; francois.paillard@chu-rennes.fr; 7 Département de Médecine Interne et Immunologie Clinique Centre de Référence des Maladies Auto-Immunes Systémiques Rares du Nord et Nord-Ouest de France (CeRAINO) CHU de Lille, F-59037 Lille, France; cecile.yelnik@chru-lille.fr; 8 U1167 Risk Factors and Molecular Determinants of Aging-Related Diseases, Inserm CHU de Lille, Lille University, F-59000 Lille, France; 9 Department of Endocrinology and Prevention of Cardiovascular Disease, Institute of Cardio Metabolism and Nutrition (ICAN), La Pitié-Salpêtrière Hospital, AP-HP, F-75005 Paris, France; valerie.carreau@aphp.fr (V.C.); eric.bruckert@aphp.fr (E.B.); antonio.gallo@univ-reunion.fr (A.G.); philippe.giral@aphp.fr (P.G.); 10 Institut Cochin, Bâtiment Faculté Inserm U1016, Cnrs UMR8104, Université de Paris Faculté de Médecine, F-75014 Paris, France; anne.philippi@inserm.fr; 11 INSERM UMRS 1166, Faculty of Medicine Pitié-Salpêtrière, Sorbonne University, F-75005 Paris, France; olivier.bluteau@aphp.fr (O.B.); alain.carrie@aphp.fr (A.C.); 12 Genetic Department, AP-HP, Hôpital Bichat, F-75018 Paris, France; 13 Department of Biochemistry and Molecular Genetics, Ambroise Paré University Hospital (APHP), Université Paris-Saclay, F-92104 Boulogne-Billancourt, France; 14 UFR (Unite de Formation et de Recherche) Simone Veil-Santé, Versailles-Saint-Quentin-en-Yvelines University, F-78180 Montigny-le-Bretonneux, France

**Keywords:** hypercholesterolemia, ADH, FCHL, apolipoprotein E, APOE gene, mutation, variant

## Abstract

Primary hypercholesterolemia is characterized by elevated LDL-cholesterol (LDL-C) levels isolated in autosomal dominant hypercholesterolemia (ADH) or associated with elevated triglyceride levels in familial combined hyperlipidemia (FCHL). Rare *APOE* variants are known in ADH and FCHL. We explored the *APOE* molecular spectrum in a French ADH/FCHL cohort of 5743 unrelated probands. The sequencing of *LDLR*, *PCSK9*, *APOB*, and *APOE* revealed 76 carriers of a rare *APOE* variant, with no mutation in *LDLR*, *PCSK9,* or *APOB*. Among the 31 *APOE* variants identified here, 15 are described in ADH, 10 in FCHL, and 6 in both probands. Five were previously reported with dyslipidemia and 26 are novel, including 12 missense, 5 synonymous, 2 intronic, and 7 variants in regulatory regions. Sixteen variants were predicted as pathogenic or likely pathogenic, and their carriers had significantly lower polygenic risk scores (wPRS) than carriers of predicted benign variants. We observed no correlation between LDL-C levels and wPRS, suggesting a major effect of *APOE* variants. Carriers of p.Leu167del were associated with a severe phenotype. The analysis of 11 probands suggests that carriers of an *APOE* variant respond better to statins than carriers of a *LDLR* mutation. Altogether, we show that the *APOE* variants account for a significant contribution to ADH and FCHL.

## 1. Introduction

Autosomal dominant hypercholesterolemia (ADH) is a major cause of premature atherosclerosis with a risk 13 times greater than all other coronary heart diseases (CHD) risk factors [1]. ADH is characterized by a selective increase in circulating low-density lipoproteins (LDL) due to reduced catabolism [2]. This increased level of LDL-cholesterol (LDL-C) in plasma since birth gives rise to tendon and skin xanthomas, arcus cornea, and vascular deposits, leading to premature CHD and death [3]. ADH is one of the most frequent monogenic diseases with a prevalence of one in 313 according to a recent meta-analysis [4]. The main ADH genes are encoding the LDL receptor (*LDLR*), apolipoprotein B (*APOB*) which is the LDL receptor protein-ligand, and proprotein convertase subtilisin/kexin type 9 (*PCSK9*) which enhances the intracellular degradation of LDL receptor [5]. The respective contributions of these three ADH-genes in 2054 French ADH patients are: *LDLR* 52%, *APOB* 3%, *PCSK9* 1%, whereas the remaining 44% of the probands had no ADH-mutation identified [6]. A polygenic origin is suggested in 36% of non-mutated patients [6,7]. These observations provide evidence for a greater level of genetic heterogeneity in ADH and the involvement of unknown genes [8]. In search of these new ADH-genes, a large ADH-affected French family with the *APOE* p.Leu167del mutation revealed it to be the fourth ADH-gene [9]. The study of 229 French ADH patients showed 1.3% likely pathogenic *APOE* variants indicating that the *APOE* gene significantly contributes to ADH [10]. Most ADH patients are treated with high-dose statins with an established efficacy for heterozygous carriers of *LDLR*, *APOB*, and *PCSK9* mutations [11]. In these cases, *APOE* p.Leu167del carriers respond better to statins, with or without ezetimibe, than ADH subjects with a *LDLR* mutation [12].

Familial combined hyperlipidemia (FCHL) is a common disorder of lipid metabolism that leads to elevated levels of very-low-density lipoprotein (VLDL), low-density lipoprotein (LDL), or both in the plasma, leading to mixed hyperlipidemia with increased total cholesterol and triglyceride levels. FCHL occurs in up to 3% of the general population and may account for one-third to one-half of familial causes of early CHD [13]. The phenotype of FCHL is highly variable among family members depending on genetic and environmental factors and may present itself as mixed hyperlipidemia, isolated hypercholesterolemia, and hypertriglyceridemia. The phenotype may also present itself as a normal serum lipid profile in combination with abnormally elevated levels of apoB. FCHL is genetically complex with variable penetrance [13]. Most cases of FCHL are considered polygenic [13], and several genes are described in FCHL [14]. *LDLR* gene mutations are reported in 19.6% of FCHL patients [15]. Some of these mutations are identified as causal to ADH indicating that ADH patients with hypertriglyceridemia may be misdiagnosed with FCHL [15]. Thus, there is a phenotypic and genetic overlap between ADH and FCHL. Variants in *APOE* are also reported in FCHL and are responsible for 3.5% of FCHL cases in a Spanish population, of which 1.4% are carriers of the *APOE* p.Leu167del variant [16].

Apolipoprotein E (apoE) is a major apolipoprotein that is synthesized primarily in the liver and controls lipoprotein metabolism. The *APOE* gene (NM_000041.4) is composed of four exons and encodes the 317 amino acid apoE precursor that matures to a 299 amino acid protein with a molecular mass of 34 kDa. ApoE is a component of chylomicrons, VLDL, the triglyceride-rich remnants of chylomicrons and VLDL, and high-density lipoprotein (HDL). It is also a co-factor for the lipoprotein lipase responsible for triglyceride hydrolysis in VLDL which enables the formation of IDL and LDL. ApoE is also present on a subset of lipoprotein (a), IDL, and LDL [17]. Additionally, it is a key factor in the regulation of lipoprotein clearance through its binding to cell-surface receptors, including LDL receptor family members such as the LDL receptor, the VLDL receptor, and the LDL receptor-related protein 1 (LRP1). ApoE also binds to cell-surface heparan sulfate proteoglycans (HSPGs) [18]. An alteration of either the structure or the function of apoE could impact the metabolism and clearance of triglyceride-rich lipoproteins and plasma lipids [17,18].

Although LDL carries few apoE proteins, the concentration and size of LDL are influenced by the common apoE isoforms E2, E3, and E4 which differ within the mature protein at amino acid positions 112 and 158. ApoE3 is considered the normal isoform and contains a cysteine residue at position 112 and an arginine residue at 158. ApoE2 with a cysteine residue at both positions is defective in LDL receptor binding and is associated with the recessive form of type III hyperlipoproteinemia [19]. ApoE4 has an arginine residue at both positions 112 and 158 and is associated with increased levels of plasma LDL-C [20]. Polymorphisms in *APOE* are associated with LDL levels in genome-wide association studies [21] and are included in the wPRS calculation [7]. Variants that give rise to apoE isoforms are *APOE4* rs429358, p.Cys130Arg and *APOE2* rs7412, p.Arg176Cys. According to frequencies given by the Genome Aggregation Database (GnomAd), sequencing of about 100,000 subjects from various disease-specific and population genetic studies results in an *APOE4* rs429358 allele frequency of 14.25% and an *APOE2* rs7412 allele frequency of 6.542% in the total GnomAd population. Thus, the approximate prevalence for the *APOE* genotypes E2/E4, E3/E3, E3/E4, and E4/E4 are 0.9%, 75.9%, 14.3%, and 2.0%, respectively.

Beyond the common *APOE* variants, rare *APOE* variants are associated with different lipid pathologies including ADH and FCHL. Therefore, we aimed to explore the molecular spectrum of *APOE* variants in a French ADH/FCHL cohort.

## 2. Results

**Figure 1:**
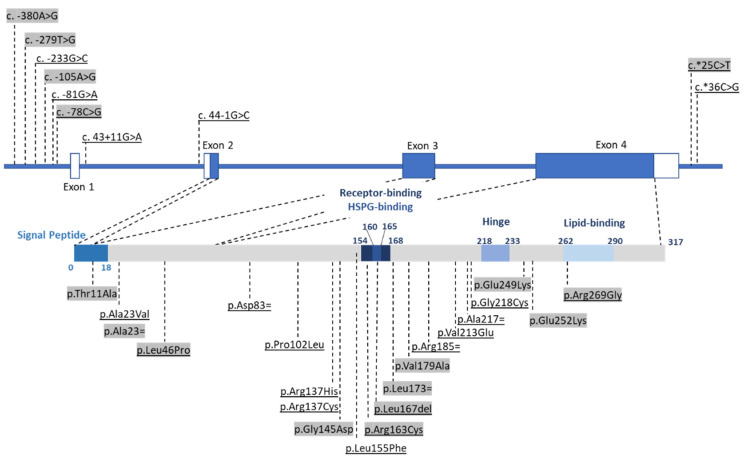
Rare *APOE* variants identified in the French ADH/FCHL cohort. Three of the four *APOE* exons encode the 317 amino acid apoE precursor. The binding site for the LDL receptor is at residues 154–168. The lipid-binding site is at residues 262–290. Between the two sites, the hinge domain is at residues 218–233. Variants are distributed on coding, intronic, promoter, and 3’UTR regions, including missense, synonymous, splicing, or regulatory variants. Variants only present in FCHL patients are highlighted in grey, and variants present in both ADH and FCHL patients are highlighted in grey and underlined.

Among 5743 probands diagnosed with primary dyslipidemia (58% ADH and 42% FCHL), we identified a total of 76 carriers of a rare *APOE* variant (53% women, 48 ± 15 years old, LDL-MoM = 1.91 ± 0.56, TG-MoM = 2.10 ± 1.65) (Table 1). None of these 76 probands carried a *LDLR*, *APOB*, or *PCSK9* variant with a deleterious or probably deleterious effect. Forty-nine patients (65%) diagnosed with ADH (55% women, 47±16 years old, LDL-MoM = 1.90 ± 0.50, TG-MoM = 1.28 ± 0.38) (Table 1) carried 21 different *APOE* variants (Figure 1, Table 2). Among the 21 variants, 3 were localized to the *APOE* promoter, 14 to exons, 2 to introns, and 2 to the 3’UTR region. Among the exonic variants, 10 were novel and not associated previously with dyslipidemia, whereas 4 were already associated with either ADH or type III hyperlipoproteinemia. Twenty-seven patients (35%) were diagnosed with FCHL (48% women, 51 ± 13 years old, LDL-MoM = 1.93 ± 0.66, TG-MoM = 3.54 ± 2.02) (Table 1) with 16 different variants (Figure 1, Table 2). Among the 16 variants, 6 were also carried by ADH probands but 10 were specific to FCHL. Only one was previously associated with primary dyslipidemia.

### 2.1. New APOE Variants in Primary Dyslipidemia

Of the 26 novel *APOE* variants (not previously reported with dyslipidemia), 12 were missense variants, 5 were synonymous substitutions, 2 were intronic, and 7 were in regulatory regions (Table 2). A large majority (21/26) were present at a higher frequency in the ADH/FCHL cohort compared to the 1148 alleles sequenced in the FREX control group that is representative of the French population, or the 152,200 alleles sequenced in GnomAD that are representative of the general population. Only two variants, c.-78C > G and p.Leu155Phe, were present at a significantly higher frequency in the ADH/FCHL cohort than in GnomAD. Moreover, the c.-78C > G variant was significantly more frequent in the ADH/FCHL cohort than in the GnomAD African/African-American population which has the highest allele frequency (Table S1). We added these data in Varsome through the activation of the PS4 ACMG criterion which identifies the prevalence of a variant in affected individuals that is significantly increased compared with the prevalence in controls. Based upon this criterion, the pathogenic prediction of c.-78C > G changed from variant of uncertain significance (VUS) to VUS/likely pathogenic (LP), and p.Leu155Phe changed from VUS/LP to LP. The c.44-1G > C variant was predicted as pathogenic because it destroyed the intron 2 acceptor splice site which may have led to the whole skipping of exon 2 or resulted in a cryptic splice site. The p.Pro102Leu variant was predicted as LP because it affected a well-conserved amino acid residue. The five synonymous variants were predicted as likely benign (LB) because they did not affect any splice site and thus might not be causative. The three 5’UTR variants nearest the gene from −78 to −105 were predicted as VUS, whereas the three farthest from the gene at −233 to −380 could not be analyzed by Varsome. In the 3’UTR, c.*25C > T was predicted to be within the miR-7704 target sequence known to be involved in tumorigenesis but not CVD [22].

### 2.2. Recurrent APOE Variants in ADH/FCHL Patients

The most frequent variant of the ADH/FCHL cohort, p.Leu167del, was carried by 14 ADH and four FCHL probands (Table 1). It was present at a significantly higher frequency in the ADH/FCHL cohort compared to the GnomAD total population (Table 2) as well as the GnomAD population with the highest allele frequency, the Latino/Admixed American population (Table S1). By adding this information in Varsome using the PS4 ACMG criterion, the pathogenic prediction changed from LP to pathogenic (P). The p.Leu46Pro variant that was previously reported in a French ADH proband [10] was carried by 12 ADH (11 heterozygotes and one homozygote) and 5 FHCL probands (Table 1). Interestingly, all carriers of the p.Leu46Pro variant were also carriers of the E4 allele due to the linkage disequilibrium between the two variants (D’ = 1.0, r^2^ = 0.266; Table S2). This variant was also reported in a dementia cohort [23]. A unique molecular event that probably occurred in the past in the E4 allele was transmitted through generations and is now reported as “ApoE4 Freiburg” [24]. The homozygote ApoE4 Freiburg carrier did not present a more severe phenotype (Table 1); thus, the transmission mode seemed to be dominant rather than semi-dominant [25]. The p.Arg163Cys variant that was previously reported in a French ADH family and two probands [10] was carried by one ADH subject who suffered from myocardial infarction at 40 years old and two FCHL subjects (Table 1). The p.Arg269Gly variant that was previously reported in one case of type IIa hyperlipidemia [9] was carried by three unrelated ADH probands and one FCHL proband (Table 1). The p.Gly145Asp variant that was previously described in a 43-year-old French patient presenting severe mixed dyslipidemia [9] was carried by two unrelated FCHL men (Table 1). It was not clear whether the variant p.Gly145Asp had an impact on the structure of apoE. The variation modified the protein net charge and thus may have altered the affinity of apoE for its receptor [10]. Interestingly, the p.Gly145Asp variant is in linkage disequilibrium with the E2 allele (D’ = 1.0, r^2^ = 0.240, Table S2).

### 2.3. Monogenic or Polygenic Dyslipidemia?

**Figure 2:**
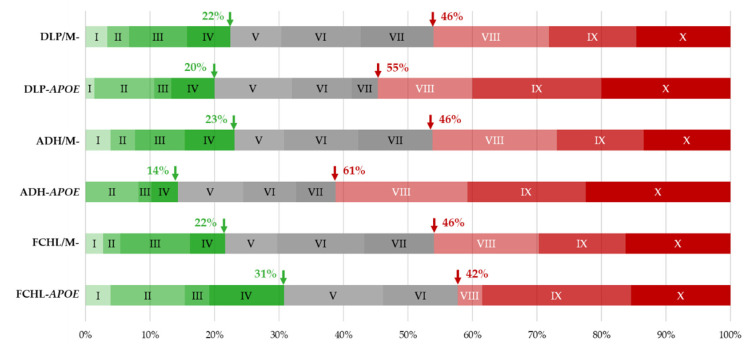
Distribution of the 12-SNP weighted polygenic risk score (wPRS) within the deciles of the Whitehall II control cohort [7]. Comparison between dyslipidemic (DLP = ADH/FCHL), ADH, or FCHL probands carrying an *APOE* rare variant or without any ADH/FCHL causative mutation (/M-). Green arrows indicate the percentage of probands with a low wPRS and a probability of monogenic DLP that gradually increases under decile V. Red arrows indicate the percentage of probands with a wPRS in the top three deciles with a high probability of polygenic DLP.

A substantial proportion of ADH/FCHL probands with no detectable mutations in *LDLR*, *APOB*, or *PCSK9* have increased LDL-C concentrations that are explainable by co-inheritance of common LDL-C-raising alleles and which are therefore of polygenic origin (19). *APOE* carriers in the ADH/FCHL cohort may have thus also presented increased LDL-C due to a polygenic origin rather than a real effect of a defective apoE. We compared the distribution of the weighted polygenic risk score (wPRS) in the ADH/FCHL, ADH, and FCHL cohorts between probands with an apoE rare variant and probands with no *LDLR*, *APOB*, *PCSK9*, or *APOE* variant (Figure 2). The proportion of probands with a high probability of polygenic dyslipidemia was increased in the cohort of *APOE* variant carriers (55% vs. 46%), whereas the probability of monogenic dyslipidemia was similar (20% vs. 22%). The difference in the proportion of probands with a high probability of polygenic dyslipidemia was more marked in the ADH cohort (61% for *APOE* variant carriers vs. 46%). This was reversed in the FCHL cohort (42% *APOE* variant carriers vs. 46%) (Figure 2).

**Figure 3:**
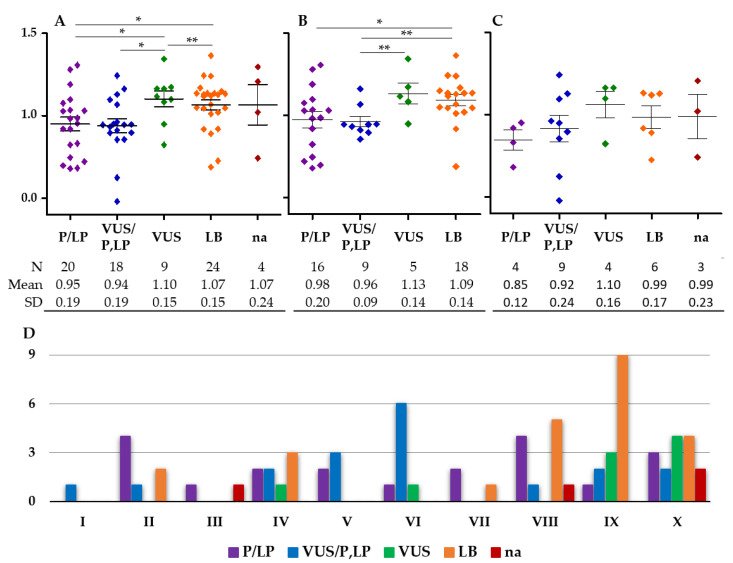
Weighted polygenic risk score (wPRS) in carriers of *APOE* variants grouped in different pathogenicity groups. The five pathogenicity groups predicted by Varsome according to the ACMG criterion are: pathogenic/likely pathogenic (P/LP); variant of uncertain significance (VUS); benign/likely benign (B/BL); predicted pathogenicity not available (na). The ADH/FCHL (A), ADH (B), and FCHL (C) cohorts are indicated above their respective plots. (D) Distribution of the variants from the five pathogenicity groups within the wPRS deciles of the Whitehall II control cohort [7]. * *p* < 0.05, ** *p* < 0.005, non-parametric Mann–Whitney test.

Conversely, the proportion of probands with a high probability of monogenic dyslipidemia was reduced in the cohort of ADH-*APOE* variant carriers (14% vs. 23%) and increased in the cohort of FCHL-*APOE* variant carriers (31% vs. 22%) (Figure 2). These observations suggest that a larger proportion of ADH cases were of polygenic origin among carriers of an *APOE* variant compared to non-carriers. Consequently, a substantial proportion of the *APOE* variants may not have been the major cause of ADH. To identify these variants, we compared the wPRS between carriers of variants grouped in the different pathogenicity groups according to Varsome classification: P/LP, VUS, and BL (Figure 3). The wPRS was significantly different among the five pathogenicity groups in the whole cohort (*p* = 0.025, Kruskal–Wallis test) (Figure 3A) and the ADH cohort (*p* = 0.022, Kruskal–Wallis test) (Figure 3B). No significant differences were observed in the FCHL cohort (Figure 3C).

**Table 1:** Description of the 76 probands with dyslipidemia.

*APOE* Variant			LDL-MoM	TC-MoM	TG-MoM	Clinical Signs	Family History	Hyperlipidemia	ApoE Isoforms	12-SNP wPRS	wPRS Decile ^b^
rs1038445539	c.-380A > G	p.?	1.67	1.44	2.07	Xanthelasma	Yes	FCHL	E3E4	0.743	III
rs1038445539	c.-380A > G	p.?	2.52	1.91	6.1	Corneal arcus	Yes	FCHL	E3E4	1.021	VIII
	c.-279G > A	p.?	1.4	1.41	5.71		Yes	FCHL	E3E4	1.207	X
-	c.-233G > C	p.?	2.00	1.71	1.30		Yes	ADH	E3E4	1.296	X
	c.-105A > G	p.?	1.9	1.78	2.56		Yes	FCHL	E3E3	1.164	X
rs766215051	c.-81G > A	p.?	2.40	1.76	1.31			ADH	E3E4	1.116	IX
rs750782549	c.-78C > G	p.?	1.57	1.41	2.55 ^a^		Yes	FCHL	E3E4	0.824	IV
rs750782549	c.-78C > G	p.?	1.59	1.60	2.04			FCHL	E3E4	1.164	X
rs750782549	c.-78C > G	p.?	1.52	1.33	1.65	Xanthelasma		ADH	E3E4	0.950	VI
-	c.43+11G > A	p.?	1.47	1.41	1.90			ADH	E3E4	1.173	X
rs770658351	c.44-1G > C	p.?	2.17	2.44	na			ADH	E3E4	0.722	II
rs144354013	c.31A > G	p.Thr11Ala	4.12	na	3.12 ^a^			FCHL	E3E4	0.950	VI
rs111833428	c.69G > A	p.Ala23=	1.41	na	5.89 ^a^			FCHL	E3E3	1.097	IX
rs776242156	c.68C > T	p.Ala23Val	1.48	1.29	0.57	CVD		ADH	E3E4	1.136	IX
rs769452	c.137T > C	p.Leu46Pro	1.59	1.48	0.96			ADH	E3E4	1.137	IX
rs769452	c.137T > C	p.Leu46Pro	1.46	1.36	0.58	Xanthoma		ADH	E4E4	1.136	IX
rs769452	c.137T > C	p.Leu46Pro	1.33	1.25	1.65			ADH	E3E4	1.012	VII
rs769452	c.137T > C	p.Leu46Pro	2.18	1.75	1.56			ADH	E3E4	1.365	X
rs769452	c.137T > C	p.Leu46Pro	1.62	1.41	0.75		Yes	ADH	E3E4	1.149	IX
rs769452	c.137T > C	p.Leu46Pro	1.40	1.25	1.70		Yes	ADH	E3E4	1.117	IX
rs769452	c.137T > C	p.Leu46Pro	1.62	1.38	1.56			ADH	E3E4	1.240	X
rs769452	c.137T > C	p.Leu46Pro	1.65	1.50	1.34			ADH	E3E4	0.919	V
rs769452	c.137T > C	p.Leu46Pro	1.53	1.86	0.77		Yes	ADH	E3E4	1.049	VIII
rs769452	c.137T > C	p.Leu46Pro	2.25	1.90	0.75		Yes	ADH	E3E4	1.045	VIII
rs769452	c.137T > C	p.Leu46Pro	1.62	1.43	1.09		No	ADH	E3E4	1.068	VIII
rs769452 ^c^	c.137T > C ^c^	p.Leu46Pro ^c^	1.75	1.46	1.42	Corneal arcus		ADH	E4E4	1.169	X
rs769452	c.137T > C	p.Leu46Pro	1.46	1.36	2.36		Yes	FCHL	E2E4	1.128	IX
rs769452	c.137T > C	p.Leu46Pro	1.72	1.61	2.86	CVD		FCHL	E3E4	1.133	IX
rs769452	c.137T > C	p.Leu46Pro	1.58	1.43	4.04 ^a^	CVD		FCHL	E3E4	0.891	V
rs769452	c.137T > C	p.Leu46Pro	1.74	1.63	2.17		Yes	FCHL	E3E4	1.120	IX
rs769452	c.137T > C	p.Leu46Pro	1.96	1.70	7.95		Yes	FCHL	E3E4	0.727	II
rs767980905	c.249C > T	p.Asp83=	1.76	1.46	1.32			ADH	E3E4	1.047	VIII
rs11083750	c.305C > T	p.Pro102Leu	2.09	1.61	1.22			ADH	E3E4	1.307	X
rs573658040	c.409C > T	p.Arg137Cys	1.61	1.43	1.21			ADH	E3E4	0.945	VI
rs11542035	c.410G > A	p.Arg137His	1.70	1.51	1.40			ADH	E3E3	0.912	V
rs267606664	c.434G > A	p.Gly145Asp	1.4	1.43	3.03			FCHL	E2E4	0.856	IV
rs267606664	c.434G > A	p.Gly145Asp	2.21	2.72	5.5 ^a^		Yes	FCHL	E3E4	0.480	I
rs1018669382	c.463 C > T	p.Leu155Phe	1.50	1.34	1.98			ADH	E3E3	1.162	X
rs769455	c.487C > T	p.Arg163Cys	1.68	1.54	1.60	CVD		ADH	E3E3	0.948	VI
rs769455	c.487C > T	p.Arg163Cys	2.47	na	10		Yes	FCHL	E3E3	0.625	II
rs769455	c.487C > T	p.Arg163Cys	1.41	1.23	2.52			FCHL	E3E4	1.129	IX
rs155726148	c.500_502delTCC	p.Leu167del	2.54	2.12	1.88	CVD		ADH	E3E3	1.280	X
rs155726148	c.500_502delTCC	p.Leu167del	2.01	1.61	1.25			ADH	E3E3	1.028	VIII
rs155726148	c.500_502delTCC	p.Leu167del	2.33	2.23	1.47			ADH	E3E3	1.030	VIII
rs155726148	c.500_502delTCC	p.Leu167del	3.51	2.66	1.16	Corneal arcus		ADH	E3E3	0.680	II
rs155726148	c.500_502delTCC	p.Leu167del	2.15	1.84	1.11			ADH	E3E3	0.747	III
rs155726148	c.500_502delTCC	p.Leu167del	3.55	2.52	1.03			ADH	E3E3	1.098	IX
rs155726148	c.500_502delTCC	p.Leu167del	2.43	2.03	1.11		Yes	ADH	E3E3	0.985	VII
rs155726148	c.500_502delTCC	p.Leu167del	2.79	2.09	1.37			ADH	E3E3	1.076	VIII
rs155726148	c.500_502delTCC	p.Leu167del	1.33	1.21	0.66			ADH	E3E3	0.920	V
rs155726148	c.500_502delTCC	p.Leu167del	2.09	1.36	1.37		Yes	ADH	E3E4	0.824	IV
rs155726148	c.500_502delTCC	p.Leu167del	2.47	2.12	0.88			ADH	E3E4	0.983	VII
rs155726148	c.500_502delTCC	p.Leu167del	1.93	1.61	1.05	Corneal arcus	Yes	ADH	E3E4	1.190	X
rs155726148	c.500_502delTCC	p.Leu167del	1.77	1.44	0.62		Yes	ADH	E3E4	1.035	VIII
rs155726148	c.500_502delTCC	p.Leu167del	1.31	2.16	1.69		Yes	ADH	E3E3	0.698	II
rs155726148	c.500_502delTCC	p.Leu167del	2.37	1.49	2.68			FCHL	E3E4	0.832	IV
rs155726148	c.500_502delTCC	p.Leu167del	1.58	1.47	2.02	CVD		FCHL	E3E3	0.683	II
rs155726148	c.500_502delTCC	p.Leu167del	1.66	1.50	2.07		Yes	FCHL	E3E4	0.921	V
rs155726148	c.500_502delTCC	p.Leu167del	3.51	2.61	3.23			FCHL	E3E3	0.952	VI
rs1239911444	c.517C > T	p.Leu173=	1.74	1.70	3.22		Yes	FCHL	E3E3	0.918	V
rs1421977676	c.536C > T	p.Val179Ala	1.65	1.57	3.05	CVD		FCHL	E3E3	na	na
rs781722239	c.555C > T	p.Arg185=	2.17	1.86	0.66	Corneal arcus		ADH	E3E3	0.689	II
-	c.638T > A ^d^	p.Val213Glu ^d^	2.51	1.99	1.07			ADH	E3E3	0.896	V
rs72654468	c.651C > T	p.Ala217=	1.54	1.42	1.13			ADH	E3E3	1.243	X
rs72654468	c.651C > T	p.Ala217=	2.08	1.69	1.85			ADH	E3E3	1.020	VIII
rs72654468	c.651C > T	p.Ala217=	1.55	1.40	1.30			ADH	E3E3	1.130	IX
-	c.652G > T	p.Gly218Cys	1.76	1.48	1.24			ADH	E3E4	0.945	VI
rs762906934	c.745G > A	p.Glu249Lys	Na	1.62	2.19	CVD		FCHL	E3E3	0.962	VI
-	c.754G > A	p.Glu252Lys	1.61	1.45	2.53		Yes	FCHL	E3E4	0.897	V
rs267606661	c.805C > G	p.Arg269Gly	2.42	1.99	2.13	CVD		FCHL	E3E4	1.243	X
rs267606661	c.805C > G	p.Arg269Gly	1.55	1.30	1.95	CVD		ADH	E4E4	0.855	IV
rs267606661	c.805C > G	p.Arg269Gly	1.81	1.50	1.51			ADH	E3E4	1.067	VIII
rs267606661	c.805C > G	p.Arg269Gly	1.71	1.52	1.43			ADH	E3E4	0.933	V
rs374329439	c.*25C > T	3’UTR variant	1.53	1.42	1.54			ADH	E3E3	1.083	IX
rs374329439	c.*25C > T	3’UTR variant	1.46	1.41	2.1			FCHL	E3E3	1.099	IX
-	c.*36C > G	3’UTR variant	1.50	1.32	1.52			ADH	E3E4	1.344	X
Median [First quartile–third quartile]			1.71 [1.54–2.17]	1.50 [1.41–1.81]	1.56 [1.21–2.36]						

na: non-available. ^a^ Triglyceride values under statin treatment. ^b^ Scores in deciles I–III have a strong probability of monogenic ADH, whereas scores in deciles VIII–X have a strong probability of polygenic hypercholesterolemia. ^c^ Homozygous carrier. ^d^ Homozygous carrier of the p.(Leu21dup) variant in *PCSK9* is known to be associated with reduced LDL-C [26].

**Table 2:** Description of the 31 *APOE* variants.

rs Number	cDNA Position (NM_000041.4)	Protein Position (NP_000032.1)	Hyperlipidemia	AF ^a^ in the ADH/FCHL Cohort	FREX Total AF ^a^	GnomAD Total AF ^a^	PolyPhen 2 ^b^	SIFT ^c^	Mutation Taster ^d^	CADD ^e^	Provean ^f^	Splice Site Affected ^g^	ACMG (Varsome) ^h^	References
rs1038445539	c.-380A > G	5’UTR variant	FCHL	0.017 (2/11,486)	0	0.005 (7/152,092)	na	na	na	7.106	na	no	na	
-	c.-279G > A	5’UTR variant	FCHL	0.009 (1/11,486)	0	0	na	na	na	5.676	na	no	na	
-	c.-233G > C	5’UTR variant	ADH	0.009 (1/11,486)	0	0	na	na	na	10.31 (top 10%)	na	no	na	
-	c.-105A > G	5’UTR variant	FCHL	0.009 (1/11,486)	0	0	na	na	DC	22.7 (top 1%)	na	no	VUS	
rs766215051	c.-81G > A	5’UTR variant	ADH	0.009 (1/11,486)	0	0.003 (5/152,130)	na	na	DC	14.13 (top 10%)	na	no	VUS	
rs750782549	c.-78C > G	5’UTR variant	ADH, FCHL	0.026 (3/11,486) ^i^	0	0.001 (2/152,116)	na	na	DC	14.91 (top 10%)	na	no	VUS	
rs770658351	c.43+11G > A	p.?	ADH	0.009 (1/11,486)	0	0	na	na	SNP	13.12 (top 10%)	na	no	VUS	
-	c.44-1G > C	p.?	ADH	0.009 (1/11,486)	0	0	na	na	DC	33 (top 0.1%)	na	Yes	P	
rs144354013	c.31A > G	p.Thr11Ala	FCHL	0.009 (1/11,486)	0	0.009 (13/151,914)	B	T	SNP	0.294	N (0.8)	no	VUS/P	
rs776242156	c.68C > T	p.Ala23Val	ADH	0.009 (1/11,486)	0	0.001 (1/152,206)	B	T	SNP	0.047	N (−0.2)	no	VUS/LP	
rs111833428	c.69G > A	p.Ala23=	FCHL	0.009 (1/11,486)	0	0.023 (35/152,212)	na	na	SNP	5.195	N (0)	no	LB	
rs769452	c.137T > C	p.Leu46Pro	ADH, FCHL	0.157 (18/11,486)	0.174 (2/1148)	0.193 (293/152,188)	P	T	DC	0.72	N (−1.1)	no	LB	[10]
rs767980905	c.249C > T	p.Asp83=	ADH	0.009 (1/11,486)	0	0.003 (4/152,218)	na	na	DC	0.615	N (0)	no	LB	
rs11083750	c.305C > T	p.Pro102Leu	ADH	0.009 (1/11,486)	0	0	PD	D	DC	23.4 (top 1%)	D (−8.7)	no	LP	
rs573658040	c.409C > T	p.Arg137Cys	ADH	0.009 (1/11,486)	0	0.002 (3/152,132)	PD	T	DC	25.8 (top 1%)	N (−2.4)	no	VUS/P	
rs11542035	c.410G > A	p.Arg137His	ADH	0.009 (1/11,486)	0	0.003(5/152,112)	P	T	SNP	22.1 (top 1%)	N (−1.0)	no	VUS/P	
rs267606664	c.434G > A	p.Gly145Asp	FCHL	0.017 (2/11,486)	0.087 (1/1148)	0.015 (22/152,152)	PD	T	DC	24.5 (top 1%)	N (0.656)	no	VUS/P	[27]
rs1018669382	c.463 C > T	p.Leu155Phe	ADH	0.009 (1/11,486) ^i^	0	0.001 (2/152,148)	B	T	SNP	5.538	N (−1.6)	no	VUS/P	
rs769455	c.487C > T	p.Arg163Cys	ADH, FCHL	0.026 (3/11,486) ^j^	0	0.643 (978/152,126)	PD	D	DC	28.4 (top 1%)	D (−4.9)	no	VUS/P	[10]
rs515726148	c.500_502delTCC	p.Leu167del	ADH, FCHL	0.157 (18/11,486) ^i^	0	0.003 (4/152,132)	na	na	SNP	na	D (−7.4)	no	LP	[9,10,16,28,29,30]
rs1239911444	c.517C > T	p.Leu173=	FCHL	0.009 (1/11,486)	0	0	na	na	DC	7.641	N (0)	no	LB	
rs1421977676	c.536T > C	p.Val179Ala	FCHL	0.009 (1/11,486)	0	0	PD	T	SNP	23.5 (top 1%)	N (−1.0)	no	VUS/P	
rs781722239	c.555C > T	p.Arg185=	ADH	0.009 (1/11,486)	0	0.009 (13/151,932)	na	na	SNP	7.192	N (0)	no	LB	
-	c.638T > A	p.Val213Glu	ADH	0.009 (1/11,486)	0	0	P	D	SNP	11.3 (top 10%)	N (−0.6)	no	VUS/P	
rs72654468	c.651C > T	p.Ala217=	ADH	0.026 (3/11,486) ^j^	0.182 (2/1,094)	0.089 (135/151,926)	na	na	SNP	6.242	N (0)	no	LB	
-	c.652G > T	p.Gly218Cys	ADH	0.009 (1/11,486)	0	0	PD	T	SNP	6.506	N (−1.4)	no	VUS/P	
rs762906934	c.745G > A	p.Glu249Lys	FCHL	0.009 (1/11,486)	0	0.001 (1/152,172)	B	T	SNP	19.7 (top 10%)	N (−1.4)	no	VUS/P	
-	c.754G > A	p.Glu252Lys	FCHL	0.009 (1/11,486)	0	0	P	D	SNP	22.2 (top 1%)	D (−2.9)	no	VUS/P	
rs267606661	c.805C > G	p.Arg269Gly	ADH, FCHL	0.035 (4/11,486)	0.087 (1/1148)	0.030 (46/152,200)	P	D	DC	23.3 (top 1%)	D (−2.9)	no	VUS/P	[10]
rs374329439	c.*25C > T	3’UTR variant	ADH, FCHL	0.017 (2/11,486)	0	0.071 (108/152,194)	na	na	SNP	5.508	na	no	VUS	
-	c.*36C > G	3’UTR variant	ADH	0.009 (1/11,486)	0	0	na	na	SNP	6.597	na	no	VUS	

^a^ AF: allele frequency in % (allele count/number), na: not available. ^b^ B: benign; PD: probably damaging; P: possibly damaging. ^c^ T: tolerated; D: deleterious. ^d^ DC: disease-causing; SNP: single nucleotide polymorphism. ^e^ Variant with a score ≥ 20 is predicted to be among the top 1% of the most deleterious substitutions in the human genome; a score ≥ 10, among the top 10%. ^f^ Variant with a score ≤ −2.5 is considered ‘deleterious’ (D) and a score ≥ 2.5 is considered “neutral” (N). ^g^ Potential effect on splicing assessed with Alamut and Human Splicing Finder; Yes: Loss of intron 2 acceptor site. ^h^ P: pathogenic; LP: likely pathogenic; VUS: variant of uncertain significance; LB: likely benign. ^i^ AF significantly higher in this ADH/FCHL cohort than in GnomAD total population. ^j^ AF significantly lower in the studied cohort than in the GnomAD total population.

In the ADH/FCHL cohort, carriers of a VUS variant presented a significantly greater mean wPRS than carriers of a P/LP, VUS/P, LP, or LB variant. Carriers of a LB variant presented a significantly greater mean wPRS than carriers of a P/LP variant (Figure 3A). In the ADH cohort, carriers of a LB variant presented a significantly higher mean wPRS than carriers of a P/LP or VUS/P,LP variant, and carriers of a VUS presented a significantly greater mean wPRS than carriers of a VUS/P,LP variant (Figure 3B). These results indicated that among carriers of VUS and LB *APOE* variants, the proportion of polygenic ADH was greater than among carriers of P/LP and VUS/P,LP variants. Thus, six VUS and six LB *APOE* variants reported here may not have been the major cause of ADH (Table 2).

The distribution of variants from the five pathogenicity groups within the wPRS deciles of the Whitehall II control cohort was significantly different between the groups (*p* = 0.003, Kruskal–Wallis test) in the ADH/FCHL cohort (Figure 3D). The VUS and LB *APOE* variants were observed more frequently in probands with a high probability of polygenic dyslipidemia compared to the P/LP and VUS/P,LP variants. Altogether, the data suggested that patients with a VUS or LB variant probably had polygenic ADH, whereas carriers of a P/LP or VUS/P,LP variant suffered from monogenic ADH due to a major effect of the *APOE* variant.

**Figure 4:**
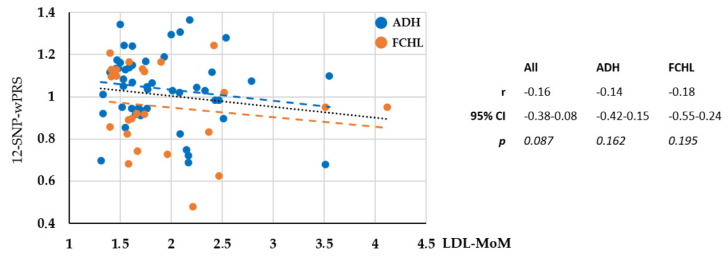
Correlation between the 12-SNP weighted polygenic risk score (wPRS) and the severity of the phenotype measured by the LDL-C. Multiple of median for LDL-C level (LDL-MoM). Non-parametric Spearman test.

We did not detect any correlation between the LDL-MoM values and the 12-SNP wPRS for the 49 ADH patients, the 27 FCHL patients, or the full cohort (Figure 4). The E4 allele accounting for a large proportion of the 12-SNP wPRS and being present at a high frequency of 33.55% in the ADH/FCHL cohort compared to 14.25% in the 200,920 alleles of the GnomAD dataset. Thus, we calculated the 10-SNP wPRS but did not detect any correlation between the LDL-MoM values and the 10-SNP wPRS. These results suggested that in the ADH/FCHL cohort, the 12 genotyped alleles that increased LDL-C, and are incorporated into the wPRS, had no significant effect on the individual level of LDL-C. Elevated LDL-C may thus have been due to a major effect of the inherited pathogenic *APOE* variant or a variant in an unidentified gene linked to dyslipidemia.

### 2.4. Genotype–Phenotype Correlation

**Figure 5:**
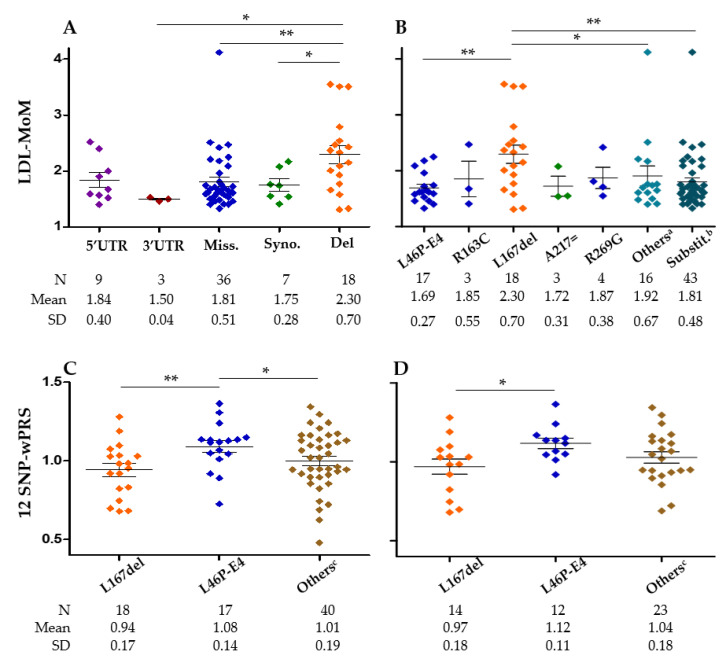
Multiple of median for LDL-C (LDL-MoM) and weighted polygenic risk score (wPRS) among carriers of different variants. (**A**) LDL-MoM in carriers of 5’UTR, 3’UTR, missense, synonymous, and deletion variants for the whole cohort. (**B**) LDL-MoM in carriers of exonic variants for the whole cohort. ^a^ Other exonic variants than the five shown in the graph because with at least three carriers, ^b^ All exonic substitutions. (**C**) wPRS in carriers of p.Leu167del, p.Leu46Pro/E4, and other variants for the whole cohort. (**D**) wPRS in the carriers of p.Leu167del, p.Leu46Pro/E4, and other variants in the ADH cohort. ^c^ Variants other than p.Leu46Pro-E4 and p.Leu167del (exonic, intronic, 5’ and 3’ UTR). * *p* < 0.05, ** *p* < 0.005, non-parametric Mann–Whitney test.

To our knowledge, no genotype–phenotype correlation has been reported among carriers of different causative variants within the *APOE* gene. The mean LDL-MoM was compared among different variants groups (Figure 5A,B). In the whole cohort, carriers of *APOE* p.Leu167del presented a significantly greater LDL-MoM than carriers of 3’UTR, missense, or synonymous variants (Figure 5A). This was also true when the p.Leu167del was compared to p.Leu46Pro/E4, other exonic variants or all the substitutions (Figure 5B). In the ADH cohort, carriers of p.Leu167del presented a significantly greater LDL-MoM than carriers of missense variants (*p* = 0.002), p.Leu46Pro/E4 (*p* = 0.008), p.Arg269Gly (*p* = 0.050), other exonic variants (*p* = 0.040), or all the substitutions (*p* = 0.002). No significant differences were observed in the FCHL cohort. Interestingly, the p.Leu46Pro/E4 carriers presented a significantly greater wPRS than p.Leu167del or all other variants combined in the whole cohort (Figure 5C). Nevertheless, no differences were observed between the three same variants groups with the 10-SNP wPRS that lacked the apoE isoform alleles: 0.99 ± 1.7, 0.91 ± 1.8, and 0.95 ± 2.0, respectively. This suggested that the E4 allele in linkage disequilibrium with the p.Leu46Pro variant supported the different 12-SNP wPRS values between the p.Leu46Pro/E4 carriers and the other variant carriers. The same observation was made in the ADH cohort (Figure 5D) but not in the FCHL cohort.

The mean TG-MoM compared among the different molecular groups showed that p.Leu167del carriers presented a significantly lesser mean TG-MoM value than all the other *APOE* variant carriers in the whole cohort (1.48 ± 0.68 vs. 2.30 ± 1.83; *p* = 0.02). However, this was not the case in the ADH or FCHL cohort. Altogether, these results suggest that the p.Leu167del *APOE* variant was associated with a monogenic form of hypercholesterolemia, increased LDL-C levels, and reduced TG levels compared to other *APOE* variants.

### 2.5. Lipid-Lowering Treatment Response

LDL-C levels with and without statin treatment were available for 11 probands of the ADH/FCHL cohort (Table 3). The observed fold-reduction of LDL-C was significantly more than estimated for FH patients carrying ADH with a mutation within the *LDLR* gene (Table 3). Most of the variants were predicted LP, VUS/P, or VUS/LP but only the p.Arg185 silent variant was predicted to be LB. Thus, it was possible that the hypercholesterolemia of the carrier of the p.Arg185 silent variant was not due to this *APOE* rare variant. Nevertheless, with the 10 other *APOE* variants, the observed fold LDL-C reduction was significantly more than expected for FH patients (2.45 ± 0. 75 vs. 1.91 ± 0.29, *p* = 0.0426). Interestingly, the only patient not presenting the expected LDL-C reduction (1.2 vs. 2.2) was the only FCHL carrier of the p.Leu167del variant and the E3E4 apoE genotype.

## 3. Discussion

In the French ADH/FCHL cohort studied here, 21 rare *APOE* variants in 49 ADH probands and 16 rare variants in 27 FCHL probands were identified, six of them being common to the two disease groups (Table 2, Figure 1). Sixteen of these rare *APOE* variants are very likely to be the major cause of the ADH/FCHL phenotype based on (1) their frequency in controls and the French ADH/FCHL cohort, (2) pathogenic prediction tools and three diagnostic lab classifications, and (3) assessment of their polygenic contribution. Although *LDLR* is still the main gene associated with primary hypercholesterolemia, our work shows that *APOE* contributes significantly, and it provides an updated full *APOE* molecular spectrum in a French ADH/FCHL cohort previously classified as mutation-negative.

In patients with ADH, triglyceride-increasing factors such as genetic and metabolic factors, diet, and *APOE* genotype could lead to the development of FCHL. Variants in the *APOE* gene may amplify the effect of these factors. Thus, according to the number or the nature of these factors, *APOE* variants could be associated with the overlapping phenotypes of FCHL, ADH, and sometimes familial dysbetalipoproteinemia when the subject is E2/E2 [31]. We, therefore, included subjects diagnosed with ADH and FCHL.

The most frequent variant in this cohort, p.Leu167del, is known as a causative mutation in ADH [9] and is the one associated with the more severe phenotype (Figure 5). The p.Leu167del variant is known to cause hypercholesterolemia in 3.1% of ADH subjects without *LDL*, *APOB*, and *PCSK9* mutations in Spain [28] and a French patient among a cohort of 229 ADH subjects [10]. The hyperLDLemia observed in the French ADH family with p.Leu167del carriers was explained by an increased LDL pool, which was the consequence of an increase in VLDL production rate and a decrease in LDL catabolism [9]. Another study showed that VLDL carrying the p.Leu167del variant produces LDL receptor downregulation resulting in increased plasma LDL-C [28]. We find in this ADH/FCHL cohort that p.Leu167del carriers are characterized by significantly higher LDL levels compared to all other *APOE* variant carriers. This is mainly due to lower LDL levels of p.Leu46Pro/E4 carriers (Figure 5B).

The variant p.Leu46Pro is the second most frequent *APOE* variant in the cohort (Table 1). When associated with the E4 isoform (ApoE Freiburg), p.Leu46Pro affects the structure and stabilization of the apoE protein [32]. Since the homozygote carrier of the ApoE Freiburg did not present a phenotype more severe than heterozygote carriers, we suggest that the disease is dominant rather than semi-dominant. This is similar to the *APOB* p.Arg3527Gln mutation for which homozygotes are reported to have cholesterol concentrations in the range of heterozygotes carriers [33]. However, they are different from carriers of *LDLR* gene mutations which present a semi-dominant disease because each allele contributes to the phenotype (OMIM nos. 143890 and 606945). However, the p.Leu46Pro variant is predicted to be benign mostly due to its relatively high frequency, 0.77% in the European Finnish population (Table S1), whereas ApoE Freiburg (p.Leu46Pro/ApoE4) is atherogenic and significantly more common among CHD patients. ApoE Freiberg is reported to be likely pathogenic in ClinVar [24] and less frequent. Its greatest allele frequency is 0.15% in the European Finnish population (Table S1).

In addition to these well-characterized variants, we identified 16 exonic missense variants, among which p.Pro102Leu and p.Val213Glu were very rare. Although the substitution p.Pro102Leu is not reported in GnomAD, p.Pro102Arg at the same position is described in a subject with hypercholesterolemia in association with the ApoE4 isoform [35]. The p.Val213Glu carrier being homozygous for the hypocholesterolemic PCSK9 L10 polymorphism (Table 1) argues for the pathogenicity of *APOE* p.Val213Glu. The p.Gly145Asp variant is associated with dyslipidemia [10,36] and modifies ApoE towards a more negative isoelectric point that may alter its affinity for the receptor. Four of the exonic variants identified in the ADH/FCHL cohort affect positively charged arginine residues. The p.Arg137Cys and p.Arg137His variants localize within the receptor-binding domain of the protein, but additional studies are needed to characterize their effects on apoE function.

The known p.Arg163Cys variant [10] is predicted to be deleterious by all tools (Table 2) and is thus classified as a pathogenic variant in Lyon’s diagnostic lab as well as in one ClinVar report. However, this variant is very frequent at 2% in the African/African-American population. This is higher than its threshold filter allele frequency by “Popmax Filtering AF” [37] of 1.98% at 95% CI. The p.Arg163Cys variant is thus classified as a benign variant in Boulogne-Billancourt’s diagnostic lab (Table S1). However, this “Popmax Filtering AF” criteria does not always seem reliable. Indeed, the p.Pro685Leu FH-causing mutation in the *LDLR* gene is recognized as pathogenic, whereas a frequency of 0.072% in the African/African-American population is greater than its “Popmax Filtering AF” of 0.019% at 95% CI.

The p.Arg269Gly variant probably changes the properties of the C-terminal helical domain of apoE resulting in altered receptor interaction with lipoproteins [9]. The variation is predicted to be deleterious by all tools (Table 2) and classified as a VUS/pathogenic variant by Varsome despite its high frequency of 0.048% in the Non-Finnish European population of GnomAD (Table S1). The allele frequency observed in the ADH/FCHL cohort for c.-78C > G and p.Leu155Phe allows a change in the pathogenic prediction from VUS and VUS/P (Table 2) to VUS/LP and LP, respectively, as in Lyon’s diagnostic lab (Table S1). These classification differences illustrate the need for additional cohort analyses and functional studies, as highlighted by Chora et al. [38]. In addition, better clinical diagnoses as proposed by Masana et al. for ADH in Spain [39] will help to build a universal consensus.

Of the 10 variants in *APOE* non-coding regions only the variant c.44-1G > C is predicted as pathogenic through possible aberrant splicing of *APOE* mRNA. Its absence in control cohorts (Table 2) and the low wPRS observed for the carrier of this variant (Table 1) are further arguments for the pathogenicity of c.44-1G > C. The variant c.*25C > T is predicted to be located within a miRNA target. Variants in the 3’UTR of cholesterol homeostasis regulatory genes such as *PCSK9* [40] are associated with modifications in cholesterol levels by miRNA regulation. However, additional studies are needed to explore if c.*25C > T affects *APOE* expression. Future functional studies in cell models expressing identified variants and RNA sequencing may be of great interest in evaluating the pathogenicity of each.

With the objective of evaluating the polygenic contribution in the ADH/FCHL cohort, we report that a greater proportion of ADH cases are polygenic among carriers of an *APOE* variant compared to ADH non-*APOE*-carriers (Figure 2). This result indicates that some *APOE* variants may not be the major cause of ADH. Furthermore, carriers of an *APOE* VUS or LB variant probably have polygenic ADH (Figure 3) and are probably not the major cause of ADH. In the *APOE*-ADH/FCHL cohort, the 12 common genotyped alleles that increase LDL-C in the weighted polygenic score (wPRS) have no significant effect on the individual level of LDL-C (Figure 4). This suggests a major effect due to the pathogenic *APOE* variant or a variant in another unidentified dyslipidemic gene.

Statins are the most used cholesterol-lowering drugs worldwide. In a small subgroup of 11 *APOE*-ADH/FCHL unrelated probands, including five p.Leu167del carriers, we report a significantly greater fold-reduction of LDL-C than estimated for FH patients who present ADH due to a mutation within the *LDLR* gene (Table 3). This improved response to statins is described in a cohort of 22 p.Leu167del Spanish carriers [12]. Our results argue for the screening of *APOE* variants in the dyslipidemia diagnosis, not only for the p.Leu167del but also for other rare variants throughout the *APOE* gene.

The main limitations of this study are the lack of functional validations and family studies to follow the segregation of the identified variants. In addition, statistical analyses are limited by the small sample size. Finally, a polygenic origin of the disease cannot be excluded in patients with a high wPRS.

## 4. Materials and Methods

### 4.1. Proband Inclusion

ADH and FCHL probands of European origin were recruited between 2012 and 2020 through the French National Research Network on Hypercholesterolemia which includes 38 clinicians from all over France. The ADH inclusion criterion was total and LDL-C values above the 90th percentile when compared to sex- and age-matched European populations [20,21]. This corresponded to a TC-MoM (see below) above 1.2 and a LDL-MoM (see below) above 1.3. The FCHL inclusion criteria were: total cholesterol and TG values above the 90th percentile when compared to sex- and age-matched European populations [41,42]. This corresponded to a TC-MoM (see below) above 1.2, and a TG-MoM (see below) above 2.0. For patients on regular treatment for whom pre-treatment values were not available, the untreated LDL-C value was estimated using the correction factors for statins ± ezetimibe medication given by a meta-analysis of 71 reports [34].

### 4.2. Molecular Analysis

DNA from peripheral blood leucocytes was amplified using the Multiplicom ADH MASTR assay v2.0 multiplexing kit (Agilent, Santa Clara, CA, USA) or libraries were prepared using Ampliseq, a SeqCapEZ Solution-Based Enrichment strategy (Roche NimbleGen Madison, WI, USA). Sequencing was performed on coding DNA sequences and flanking introns (exon padding +/−30 bp) of the *LDLR*, *PCSK9*, *APOB*, and *APOE* genes and SNPs included in the wPRS as described [43,44].

### 4.3. Variant Nomenclature

Variants were designated according to the Human Genome Variation Society recommendations (HGVS; https://www.hgvs.org/mutnomen, accessed on 6 November 2021). cDNA was numbered from +1 for A in the ATG translation initiation codon of the reference sequence (NM_000041.4). Amino acid residues were numbered from +1 for the initiating methionine of the protein sequence (NP_000032.1). Hence, 18 was added to the original numbering for ApoE corresponding to the 18 residues forming the signal peptide.

### 4.4. In Silico Variant Analyses

The causal effect of each variant was estimated with in silico prediction tools included in Alamut Visual version 2.15 (PolyPhen-2, SIFT, Mutation taster) (https://www.sophiagenetics.com/platform/alamut-visual-plus/, accessed on 6 November 2021) in addition to Provean (https://provean.jcvi.org, accessed on 6 November 2021) and CADD score (https://cadd.gs.washington.edu/snv, accessed on 6 November 2021). The potential effect of variants on splicing was assessed using Alamut Visual version 2.15 (MaxEntScan, NNSPLICE, GeneSplicer, ESE tools) and Human Splicing Finder (http://www.umd.be/hsf/, accessed on 6 November 2021). The frequency of variants in a control group representative of the French population was taken from the French Exome Project database (FREX; https://www.france-genomique.org/bases-de-donnees/frex-the-french-exome-project-database/, accessed on 6 November 2021). Variant frequencies in the general population were taken from the Genome Aggregation Database (gnomAD-v3.1.1; https://gnomad.broadinstitute.org/, accessed on 6 November 2021). ClinVar (https://www.ncbi.nlm.nih.gov/clinvar/, accessed on 6 November 2021), the Leiden Open Variation Database (LOVD; https://www.lovd.nl/, accessed on 6 November 2021), and the Human Gene Mutation Database (HGMD; http://www.hgmd.cf.ac.uk/, accessed on 6 November 2021) were used to search for variants previously reported in human diseases. The MicroRNA Target Prediction Database was also used (miRDB; http://mirdb.org/, accessed on 6 November 2021).

### 4.5. Variant Classifications

Variants were classified according to the American College of Medical Genetics and the Association of Medical Pathologists (ACMG) guidelines [45] given by Varsome (https://varsome.com, accessed on 6 November 2021). This was applied to segregation and allelic in-house data of each diagnostic center (Lyon, Boulogne-Billancourt, Paris, France) and population allelic frequencies in GnomAD (http://gnomad.broadinstitute.org/, accessed on 6 November 2021).

### 4.6. Multiple of Median for Total Cholesterol, LDL-C, and Triglyceride Level Calculation

The multiple of median (MoM) for the total cholesterol (TC-MoM), LDL-C (LDL-MoM), and triglyceride (TG-MoM) values measured the deviation from the mean of a reference population of individual values. It allowed the comparison of lipid levels adjusted for age and gender using data from a French population of children [41] and a Dutch population of adults [42]. The MoMs are a ratio determined by the following: LDL-/TC-/TG-MoM = (LDL-C/TC/TG of the patient)/(LDL-C/TC/TG of the 50th percentile of his sex and age class)

### 4.7. Weighted Polygenic Risk Score (wPRS)

For each individual, the wPRS was calculated using the weighted sum of the risk allele for the 12 SNPs (alleles increasing LDL-C) and compared to those of 3020 normocholesterolemic men and women of European ancestry from the UK Whitehall II (WHII) cohort study [7]. The 10-SNP wPRS excluded the contribution of the ApoE isoform alleles: 12-SNP wPRS −0.2 for E4E4, −0.1 for E3E4, and +0.2 for E2E4.

### 4.8. Statistics

Statistical analyses were performed with JMP software (SAS Institute Inc., Cary, NC, USA) and GraphPad Prism® software. The non-parametric Mann–Whitney U test assessed differences between two groups. The non-parametric Kruskal–Wallis test assessed differences among more than two groups. The Spearman r test assessed the correlation between two variables. The non-parametric Wilcoxon matched-pairs test evaluated differences between the observed reduction of LDL-C levels after treatment and the expected reduction. *p* values ≤ 0.05 were considered statistically significant. Pairwise linkage disequilibria between the most frequent *APOE* variants having minor allele frequencies > 0.01% in the 76 index cases from the cohort were estimated by using Haploview 4.2 [46] and PLINK [47].

## 5. Conclusions

Through the sequencing of *APOE* in patients diagnosed with primary dyslipidemias without a mutation in the *LDLR*, *APOB*, or *PCSK9* genes, we report a substantial number of rare variant carriers. However, the complex role of the ApoE in lipid homeostasis and the limited number of subjects make the interpretation of variant pathogenicity difficult. Although additional factors such as family segregation and functional studies may influence our interpretation, we conclude that screening of *APOE* should be included in routine diagnoses for ADH and FCHL to improve the prognosis and care management of patients and their families.

## Figures and Tables

**Table 3:** LDL-C reduction under statins.

*APOE* Variant			Pathogenic Prediction ^a^	Gender	Age ^b^	LDL-C without Preatment ^c^	Treatment	Age ^d^	LDL-C under Preatment ^c^	Estimated Reduction ^e^	Observed Reduction
rs776242156	c.68C > T	p.Ala23Val	VUS/LP	M	43	5.17	Atorvastatin 20	46	1.42	1.8	3.6
rs11542035	c.410G > A	p.Arg137His	VUS/P	F	61	6.45	Simvastatin 20	62	3.06	1.6	2.1
rs769455	c.487C > T	p.Arg163Cys	VUS/P	M	40	5.88	Atorvastatin 80 Ezetimibe 10	41	1.69	2.5	3.5
rs155726148	c.500_502delTCC	p.Leu167del	LP	M	69	7.24	Atorvastatin 20	70	2.74	1.8	2.6
rs155726148	c.500_502delTCC	p.Leu167del	LP	F	38	9.44	Atorvastatin 80	56	3.59	2.2	2.6
rs155726148	c.500_502delTCC	p.Leu167del	LP	F	31	6.18	Atorvastatin 80	32	5.20	2.2	1.2
rs155726148	c.500_502delTCC	p.Leu167del	LP	M	31	7.55	Simvastatin 20 Ezetimibe 10	38	2.87	1.8	2.6
rs155726148	c.500_502delTTC	p.Leu167del	LP	M	20	6.99	Rosuvastatin 5	27	3.74	1.8	1.9
rs781722239	c.555C > T	p.Arg185=	LB	M	65	7.81	Atorvastatin 20	65	4.29	1.8	1.8
rs267606661	c.805C > G	p.Arg269Gly	VUS/P	M	51	5.73	Atorvastatin 20	58	3.18	1.8	1.8
rs267606661	c.805C > G	p.Arg269Gly	VUS/P	M	59	6.33	Atorvastatin 10	59	2.49	1.6	2.5
	Mean									1.90	2.39
	SD									0.28	0.74
	*Wilcoxon matched-pairs test*									*p* = 0.0426	

^a^ ACMG criteria from Varsome (Table 2); P: pathogenic; LP: likely pathogenic; VUS: variant of uncertain significance; LB: likely benign. ^b^ Age at lipid measurement without treatment. ^c^ mmol/L. ^d^ Age at lipid measurement under treatment. ^e^ Correction factors were obtained by the meta-analysis of 71 studies [34].

## Data Availability

Data are available on request to the corresponding author.

## Supplementary Materials

The following supporting information can be downloaded at: https://www.mdpi.com/article/10.3390/ijms23105792/s1.
