# Supplementary material for: APOE Molecular Spectrum in a French Cohort with Primary Dyslipidemia
Source: Int J Mol Sci. 2022 May 21;23(10):5792. doi: 10.3390/ijms23105792 (PMC9145810; doi:10.3390/ijms23105792)
Supplement: Supplementary file 1 [file ijms-23-05792-s001.zip › ijms-1719092-supplementary.pdf]

International Journal of *Molecular Sciences*

Supplementary Materials:

Table S1: Frequency and ACGM classification of the 31 *APOE* variants.
Table S2: Pairwise linkage disequilibrium for the most frequent *APOE* variants with minor allele frequency >0.01 % in the 76 index cases of the cohort.

Table S1: Frequency and ACGM classification of the 31 *APOE* variants.

rs number	cDNA position (NM_000041.4)	Protein position (NP_000032.1)	AF in the ADH/FCHL cohort [a]	gnomAD total AF [a]	gnomAD highest AF [a] (population [b])	ACMG (Varsome) [e]	Diagnostic report in Lyon [e]	Diagnostic report in Boulogne Billancourt [e]	Diagnostic report in Paris [e]
rs1038445539	c.-380A>G	5′UTR variant	0.017 (2/11.486)	0.005 (7/152 092)	0.026 (L/AA. 4/15260)	na	VUS	na	VUS
-	c.-279G>A	5′UTR variant	0.009 (1/11.486)	0	na	na	VUS	na	na
-	c.-233G>C	5′UTR variant	0.009 (1/11.486)	0	na	na	VUS	na	na
-	c.-105A>G	5′UTR variant	0.009 (1/11.486)	0	na	VUS	VUS	na	VUS
rs766215051	c.-81G>A	5′UTR variant	0.009 (1/11.486)	0.003 (5/152.130)	0.005 (Af/Af-Am. 2/41432)	VUS	VUS	na	VUS
rs750782549	c.-78C>G	5′UTR variant	0.026 (3/11.486) [d.e]	0.001 (2/152.116)	0.003 (Af/Af-Am. 1/41416)	VUS	VUS/LP	na	VUS
rs770658351	c.43+11G>A	p.?	0.009 (1/11.486)	0	na	VUS	VUS	na	VUS
-	c.44-1G>C	p.?	0.009 (1/11.486)	0	na	P	LP	na	P
rs144354013	c.31A>G	p.Thr11Ala	0.009 (1/11.486)	0.009 (13/151.914)	0.072 (L/AA. 11/15248)	VUS/P	VUS	na	VUS
rs776242156	c.68C>T	p.Ala23Val	0.009 (1/11.486) [d]	0.001 (1/152.206)	0.002 (Af/Af-Am. 1/41442)	VUS/LP	LP	VUS	na
rs111833428	c.69G>A	p.Ala23=	0.009 (1/11.486)	0.023 (35/152.212)	0.043 (NFE. 29/68042)	LB	VUS	na	na
rs769452	c.137T>C	p.Leu46Pro	0.157 (18/11.486)	0.193 (293/152.188)	0.772 (FE. 82/10616)	LB	VUS	B	LB
rs767980905	c.249C>T	p.Asp83=	0.009 (1/11.486)	0.003 (4/152.218)	0.004 (NFE. 3/68032)	LB	VUS	na	na
rs11083750	c.305C>T	p.Pro102Leu	0.009 (1/11.486)	0	na	LP	VUS	na	na
rs573658040	c.409C>T	p.Arg137Cys	0.009 (1/11.486)	0.002 (3/152.132)	0.003 (NFE. 2/67984)	VUS/P	VUS	na	VUS
rs11542035	c.410G>A	p.Arg137His	0.009 (1/11.486))	0.003(5/152.112)	0.006 (NFE. 4/67996)	VUS/P	VUS	na	na
rs267606664	c.434G>A	p.Gly145Asp	0.017 (2/11.486)	0.015 (22/152.152)	0.025 (NFE. 17/67992)	VUS/P	VUS	na	VUS
rs1018669382	c.463 C>T	p.Leu155Phe	0.009 (1/11.486) [d]	0.001 (2/152.148)	0.005 (Af/Af-Am. 2/41460)	VUS/P	LP	na	na
rs769455	c.487C>T	p.Arg163Cys	0.026 (3/11.486) [f]	0.643 (978/152.126)	2.097 (Af/Af-Am. 869/41444)	VUS/P	P	B	VUS
rs515726148	c.500_502delTCC	p.Leu167del	0.157 (18/11.486) [d.e]	0.003 (4/152.132)	0.013 (L/AA. 2/15268)	LP	LP	P	LP
rs1239911444	c.517C>T	p.Leu173=	0.009 (1/11.486)	0	na	LB	VUS	na	na
rs1421977676	c.536T>C	p.Val179Ala	0.009 (1/11.486)	0	na	VUS/P	VUS	na	na
rs781722239	c.555C>T	p.Arg185=	0.009 (1/11.486)	0.009 (13/151.932)	0.019 (NFE. 13/67918)	LB	VUS	LB	na
-	c.638T>A	p.Val213Glu	0.009 (1/11.486)	0	na	VUS/P	VUS	VUS	na
rs72654468	c.651C>T	p.Ala217=	0.026 (3/11.486) [f]	0.089 (135/151.926)	0.144 (L/AA. 22/15268)	LB	LB	B	na
-	c.652G>T	p.Gly218Cys	0.009 (1/11.486)	0	na	VUS/P	VUS	na	VUS
rs762906934	c.745G>A	p.Glu249Lys	0.009 (1/11.486) [d]	0.001 (1/152.172)	0.002 (Af/Af-Am. 1/41464)	VUS/P	LP	na	na
-	c.754G>A	p.Glu252Lys	0.009 (1/11.486)	0	na	VUS/P	VUS	na	VUS
rs267606661	c.805C>G	p.Arg269Gly	0.035 (4/11.486)	0.030 (46/152.200)	0.049 (NFE. 33/68022)	VUS/P	VUS	LB	na
rs374329439	c.*25C>T	3′UTR variant	0.017 (2/11.486)	0.071 (108/152.194)	0.317 (FE. 20/10622)	VUS	VUS	B	VUS
-	c.*36C>G	3′UTR variant	0.009 (1/11.486)	0	na	VUS	VUS	VUS	na

[a] AF: Allele frequency in % (allele count/number). na: not available. [b] L/AA: Latino/Admixed american; Af/Af-Am: African/African-American; NFE: Non-Finnish European; FE: Finnish European. [c] P: Pathogenic; LP: Likely Pathogenic; VUS: Variant of Uncertain Significance; LB: Likely Benign; B: Benign. [d] AF significantly higher in the studied cohort than in the GnomAD total population. [e] AF significantly higher in the studied cohort than in the GnomAD population with the highest AF. [f] AF significantly lower in the studied cohort than in GnomAD total population.

Table S2: Pairwise linkage disequilibrium for the most frequent *APOE* variants with minor allele frequency >0.01 % in the 76 index cases of the cohort.

r^2 / D′	rs750782549 c.-78C>G	rs769452 p.Leu46Pro	rs429358 (ε4) p.Cys130Arg	rs267606664 p.Gly145Asp	rs769455 p.Arg163Cys	rs515726148 p.Leu167del	rs7412 (ε2) p.Arg176Cys	rs267606661 p.Arg269Gly	rs374329439 c.*25C>T
rs750782549 c.-78C>G		0.003	0.040	0.000	0.000	0.003	0.000	0.001	0.000
rs769452 p.Leu46Pro	1.0		**0.266**	0.002	0.003	0.018	0.012	0.004	0.002
rs429358 (ε4) p.Cys130Arg	1.0	1.0		0.026	0.010	0.068	0.026	0.054	0.007
rs267606664 p.Gly145Asp	1.0	1.0	1.0		0.000	0.002	**0.240**	0.000	0.000
rs769455 p.Arg163Cys	1.0	1.0	1.0	1.0		0.003	0.000	0.001	0.000
rs515726148 p.Leu167del	1.0	1.0	1.0	1.0	1.0		0.002	0.004	0.002
rs7412 (ε2) p.Arg176Cys	1.0	1.0	1.0	1.0	1.0	1.0		0.000	0.000
rs267606661 p.Arg269Gly	1.0	1.0	1.0	1.0	1.0	1.0	1.0		0.000
rs374329439 c.*25C>T	1.0	1.0	1.0	1.0	1.0	1.0	1.0	1.0	

D′ values are given below and r^2 above the grey diagonal. Association of **minor allele with minor allele is indicated in bold**.
